# Genomic Epidemiology of Multidrug-Resistant Nontyphoidal Salmonella in Young Children Hospitalized for Gastroenteritis

**DOI:** 10.1128/spectrum.00248-21

**Published:** 2021-08-04

**Authors:** Pei Yee Woh, May Pui Shan Yeung, William Bernard Goggins, Norman Lo, Kam Tak Wong, Viola Chow, Ka Yee Chau, Kitty Fung, Zigui Chen, Margaret Ip

**Affiliations:** a Jockey Club School of Public Health and Primary Care, Faculty of Medicine, The Chinese University of Hong Kong, Hong Kong Special Administrative Region; b Department of Microbiology, Faculty of Medicine, The Chinese University of Hong Kong, Prince of Wales Hospital, Hong Kong Special Administrative Region; c Department of Pathology, Alice Ho Miu Ling Nethersole Hospital, Tai Po, Hong Kong Special Administrative Region; d Department of Pathology, United Christian Hospital, Kowloon, Hong Kong Special Administrative Region; Houston Methodist Hospital

**Keywords:** nontyphoidal *Salmonella*, gastroenteritis, whole-genome sequencing, multidrug resistance, children

## Abstract

Nontyphoidal Salmonella (NTS) gastroenteritis in children remains a significant burden on health care and constitutes a majority of all admissions for Salmonella infections in public hospitals in Hong Kong. In this prospective study, 41% of 241 children hospitalized with gastroenteritis from three public hospitals during 2019 were culture confirmed to have NTS infection. These Salmonella isolates were whole-genome sequenced and *in silico* predicted for their serovars/serotypes using the Salmonella *In Silico* Typing Resource (SISTR) and SeqSero1, and the antimicrobial resistance (AMR) genes were determined. Phylogenetic analysis revealed three major clades belonging to Salmonella enterica serovar Enteritidis sequence type 11 (ST11) (43%), multidrug-resistant (MDR) *S.* Typhimurium ST19 (12%) and its monophasic variant ST34 (25%), and mostly singletons of 15 other serovars. MDR *S.* Typhimurium and its variant were more common in infants <24 months of age and possessed genotypic resistance to five antimicrobial agents, including ampicillin (A), chloramphenicol (C), aminoglycosides (Am), sulfonamides (Su), and tetracyclines (T). Older children were more often infected with *S.* Enteritidis, which possessed distinct genotypic resistance to AAmSu and fluoroquinolones. In addition, 3% of the isolates possessed extended-spectrum beta-lactamase (ESBL) CTX-M genes, while one isolate (1%) harboring the carbapenemase gene *bla*_NDM-1_ was identified. Our findings provide a more complete genomic epidemiological insight into NTS causing gastroenteritis and identify a wider spectrum of determinants of resistance to third-generation beta-lactams and carbapenems, which are often not readily recognized. With high rates of multidrug-resistant NTS from studies in the Asia-Pacific region, the rapid and reliable determination of serovars and resistance determinants using whole-genome sequencing (WGS) is invaluable for enhancing public health interventions for infection prevention and control.

**IMPORTANCE** Nontyphoidal Salmonella (NTS) gastroenteritis is a foodborne disease with a large global burden. Antimicrobial resistance (AMR) among foodborne pathogens is an important public health concern, and multidrug-resistant (MDR) Salmonella is prevalent in Southeast Asia and China. Using whole-genome sequencing, this study highlights the relationship of the MDR Salmonella serotypes and the diverse range of Salmonella genotypes that contaminate our food sources and contribute to disease in this locality. The findings update our understanding of Salmonella epidemiology and associated MDR determinants to enhance the tracking of foodborne pathogens for public health and food safety.

## INTRODUCTION

Nontyphoidal Salmonella (NTS) remains an important etiology of diarrhea and is responsible for an estimated 197.35 million episodes (95% uncertainty interval [UI], 127.37 million to 299.20 million), with 84,799 deaths (95% UI, 46,201 to 144,935) annually, according to the Global Burden of Diarrheal Disease study in 2016 (1). The burden disproportionately affects children under 5 years of age, and while most cases are mild, our review of 10-year admissions of patients with Salmonella infections from public hospitals of Hong Kong revealed that 88.1% of the 4,828 admissions were children aged ≤15 years (2). NTS gastroenteritis among children thus remains a significant burden on health care. Although antibiotics are not routinely recommended except in cases with invasive complications or bacteremia (3), our previous study showed that substantially longer stays and higher costs were associated with hospitalization with fluoroquinolone-resistant Salmonella spp. than with the cohort with infections caused by susceptible strains (4). Thus, interventions that reduce its burden or reduce antimicrobial resistance (AMR) may reduce the morbidity and health care costs of these infections.

Whole-genome sequencing (WGS)-based prediction of serovars is rapid and provides genotypic details for outbreak identification, source tracking, and diagnostic and epidemiological surveillance. With over 2,600 reported serovars (serotypes) to date (5), most diagnostic laboratories resort to only serogroup determination using polyclonal antibodies, without serovar information that facilitates interventions for outbreak investigations.

Accumulating evidence from performance comparisons of *in silico* serovar prediction tools (6–8) revealed that the Salmonella *In Silico* Typing Resource [SISTR] (9) has higher concordance than other methods, including SeqSero1 (10), SeqSero2 (11), multilocus sequence typing (MLST) (12), or metric-oriented sequence typing (MOST) (13). In addition, WGS allows the prediction of AMR genes, which further enhances the differentiation between clades of the same serotype. For example, two clades of Salmonella enterica serovar Typhimurium were described in Hong Kong (14), one of which was multidrug resistant (MDR) to ampicillin (AM), chloramphenicol (CHL), and trimethoprim. Recently, we identified a high prevalence of extended-spectrum beta-lactamase (ESBL)- and carbapenemase-producing *Enterobacteriaceae* from food animal products in our local wet markets (15). Such ESBLs and carbapenem resistance have been increasingly reported in Salmonella spp. from clinical cases, animals, and food samples (16, 17). This highlights the importance of the monitoring and tracking of AMR in association with specific Salmonella serotypes.

In this study, we sought to use WGS to describe the epidemiology of NTS isolated from children hospitalized with Salmonella gastroenteritis from three acute-care hospitals in Hong Kong in 2019. The study infers detailed epidemiological insights into the Salmonella serovars, AMR, and characteristics of the common NTS serotypes causing gastroenteritis requiring hospitalization in children.

## RESULTS

### Concordance of serotypes and *in silico* serovar prediction.

Salmonella serovars were predicted by SISTR and SeqSero1, and details of the serovars are listed in Data Set S1 in the supplemental material. One strain (isolate P144_06/19) did not have sufficient sequence reads for identification and was excluded from further analysis. SISTR predicted serovars with the full antigenic formula for all the strains, while SeqSero1 predicted only 95.0% with the full antigenic formula (Table S1). SeqSero1 could not distinguish five isolates due to the provision of only partial antigenic formulas lacking H1 or H2 antigens and for two isolates gave multiple serovar assignments to S. enterica serovar Goldcoast or *S*. Brikama (isolate A030_06/19) (Table 1) and *S*. Javiana or Salmonella serotype II 9,12:l,z28:1,5 (isolate A161_10/19) (Table 1). Disagreements on the traditional serogroup results were noted for three strains, whereby two strains belonged to groups C and P by both SISTR and SeqSero1, whereas the serogroup determination by agglutination misidentified both strains as belonging to group B, and a further isolate could not be serotyped by the traditional method (Table S2).

**TABLE 1:** Isolates with discordant serotype predictions between SISTR and SeqSero1

Isolate ID	Serotype predicted by:	
	SISTR	SeqSero1^*a*^
P322_08/19	*S.* Typhimurium 4:i:1,2	Monophasic variant *S.* Typhimurium 4:i:–
P384_10/19	*S.* Stanley 4:d:1,2	NA 4:d:–
A006_05/19	*S.* Concord 7:l,v:1,2	NA 7:–:1,2
A013_06/19	*S.* Mgulani 38:i:1,2	NA 38:–:1,2
A030_06/19	*S.* Goldcoast 8:r:l,w	*S.* Goldcoast or *S.* Brikama 8:r:l,w^*b*^
A038_06/19	*S.* Typhimurium 4:i:1,2	NA 4:–:–
A081_08/19	*S.* Saintpaul 4:e,h:1,2	NA 4:–:1,2
A161_10/19	*S.* Javiana 9:l,z28:1,5	Salmonella II 9,12:l,z28:1,5 or *S.* Javiana 9,12:l,z28:1,5^*b*^

a NA, not applicable.

b Multiple serovar predictions sharing the same antigenic formula.

### Antimicrobial resistance and concordance between phenotypic and genotypic methods.

The most common phenotypic resistances were to ampicillin (76.0%), tetracycline (T) (60.4%), ciprofloxacin (CIP) (56.0%), chloramphenicol (25.0%), and trimethoprim-sulfamethoxazole (SXT) (23.0%). Seven percent of isolates were resistant to third-generation cephalosporins (cefotaxime [CTX]/ceftriaxone [CRO]), and 1.9% were resistant to azithromycin (AZM) (*n* = 1/52). No strains were resistant to meropenem (MEM). The individual isolate-level data for antibiotic phenotypic and genotypic resistance are shown in Data Set S2.

The WGS-based genotypic analysis revealed 46 unique resistance genes (shown in Data Set S3). Genes for CTX-M-type extended-spectrum beta-lactamases (ESBLs) were present in six isolates, with *bla*_CTX-M-55_ (*n* = 4), *bla*_CTX-M-64_ (*n* = 1), and *bla*_CTX-M-65_ (*n* = 1) in *S*. Typhimurium (*n* = 1) or its monophasic variant (*n* = 3) or in *S.* Enteritidis (*n* = 2). One *S*. Typhimurium isolate carried the carbapenemase gene *bla*_NDM-1_. One *S.* Enteritidis isolate carried the colistin resistance gene *mcr-1*.

For fluoroquinolone resistance, both quinolone resistance-determining region (QRDR) mutations (*gyrA* and *parC*) and plasmid-mediated quinolone resistance (PMQR) genes [*aac(6′)-Ib-cr*, *qnrS1*, and *oqxAB*] were identified in this study. *S*. Derby (*qnrS1 parC*), *S.* Enteritidis (*qnrS1 gyrA*), *S*. Goldcoast (*qnrS1 parC*), *S*. Typhimurium monophasic variant 4,[5],12:i:– [*aac(6′)-Ib-cr qnrS1 oqxAB gyrA parC*], and *S*. Typhimurium [*aac(6′)-Ib-cr qnrS1 oqxAB gyrA parC*] carried both QRDR mutations and PMQR genes (Table S3). Of the 46 distinct *tet* alleles, only 3 were identified, including the efflux pump-encoding *tetA* (25%) and *tetB* (26%) genes and the ribosomal protection protein-encoding *tetM* gene (12%); the latter was present in isolates in addition to *tetA* and *tetB*.

Overall, phenotypic resistance was highly correlated with the presence of known resistance determinants, with genotype agreeing with phenotype for 486 of 502 phenotypic tests for all nine antimicrobials from six different classes. This resulted in an overall concordance between the two of 96.8%. In total, 174 of the 502 antibiotic tests indicated resistance, and associated genes were predicted to cause resistance to all antimicrobials tested except meropenem. This resulted in an overall sensitivity of 96.6% (168/174) (Table 2). Among the 328 phenotypic susceptibility test results, there were 10 occasions where resistance genes were detected by WGS. Among these resistance genes, 8 were related to ciprofloxacin, and 2 were related to chloramphenicol. This resulted in an overall specificity of 97.0% (318/328) (Table 2).

**TABLE 2:** Genotype and phenotype comparison of Salmonella spp. (*n* = 100)^*a*^

Antimicrobial class	Antibiotic (no. of isolates tested)	No. of isolates				Sensitivity (%)	Specificity (%)
		Resistant phenotype		Susceptible phenotype			
		Resistant genotype (TP)	Susceptible genotype (FN)	Resistant genotype (FP)	Susceptible genotype (TN)		
Beta-lactams	AM (100)	75	1	0	24	98.7	100
	CTX-CRO (100)	7	0	0	93	100	100
	MEM (4)	0	0	0	4	—^*b*^	100
Folate pathway inhibitors	SXT (100)	21	2	0	77	91.3	100
Macrolides	AZM (52)	1	0	0	51	100	100
Phenicols	CHL (48)	10	2	2	34	83.3	94.4
Quinolones	CIP (50)	25	1	8	16	96.2	66.7
Tetracyclines	T (48)	29	0	0	19	100	100

Total		168	6	10	318	96.6	97.0

a TP, true positive; FN, false negative; FP, false positive; TN, true negative [sensitivity = TP/(TP + FN); specificity = TN/(TN +FP)]. AM, ampicillin; CTX-CRO, cefotaxime-ceftriaxone; MEM, meropenem; SXT, trimethoprim-sulfamethoxazole; AZM, azithromycin; CHL, chloramphenicol; CIP, ciprofloxacin; T, tetracycline.

b Sensitivity and specificity were not computed because the resistant phenotype was constant.

### MLST and phylogenetic analysis.

**FIG 1:**
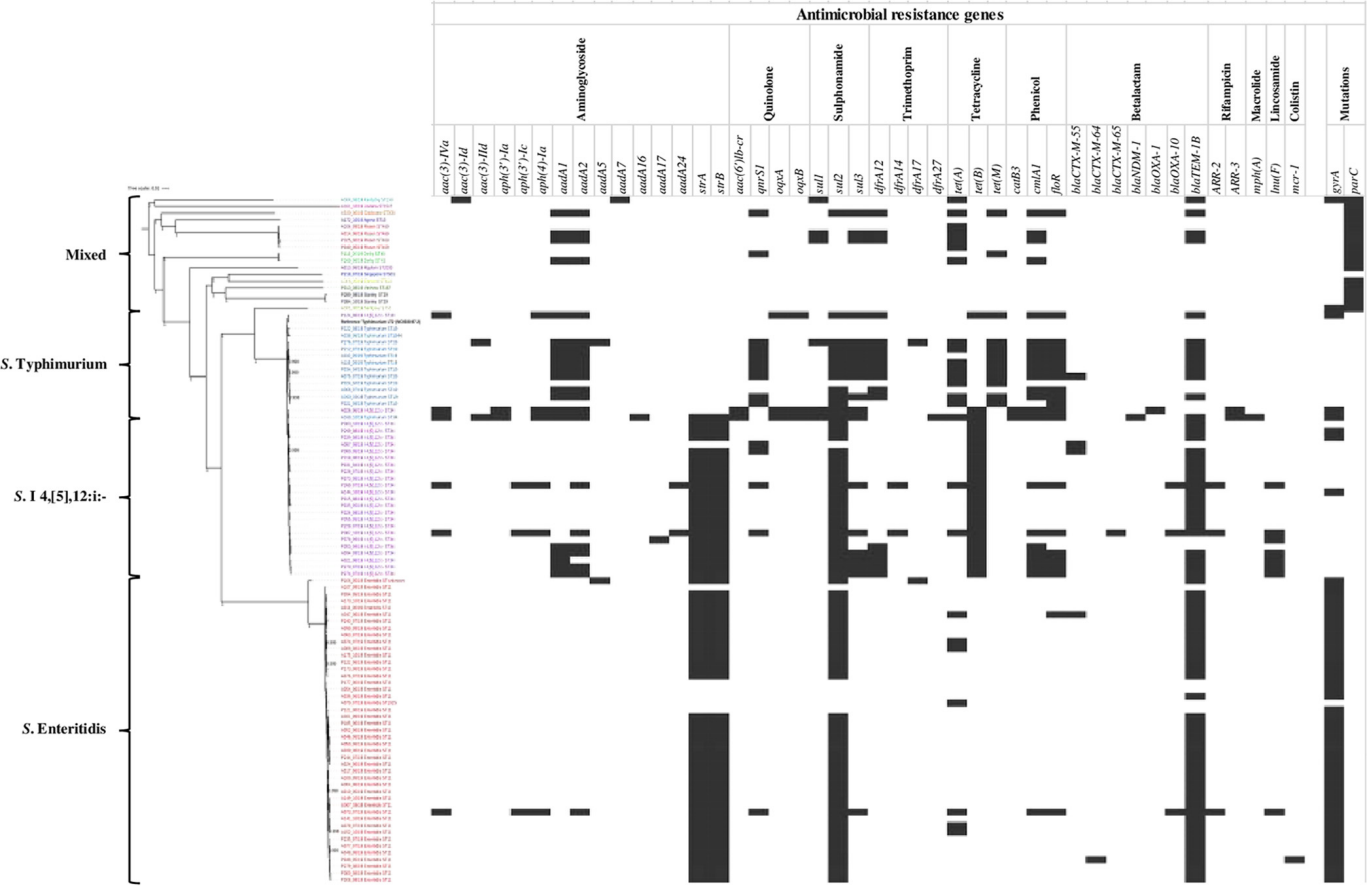
SNP phylogenetic tree of Salmonella enterica constructed using CSI Phylogeny. Black boxes indicate the presence of an AMR gene and, for *gyrA* and *parC*, inferred gene mutations as identified using CGE PointFinder. Serovars are labeled in color (isolate identifier_sampling date_serotype_sequence type [ST]). *S*. Typhimurium LT2 (RefSeq accession no. NC_003197.2) is the reference genome.

MLST 2.0 and SISTR core-genome MLST (cgMLST) gave identical MLST types for all Salmonella isolates, except for one that has an unknown sequence type (ST) (Table 3). The three predominant types were *S.* Enteritidis ST11 (*n* = 43), monophasic variant *S*. Typhimurium ST34 (*n* = 25), and *S.* Typhimurium ST19 (*n* = 12). The percentage of reference genomes covered by all isolates was 77.6%, with 3,772,527 nucleotide positions present in all analyzed genomes. The phylogenetic tree revealed three major clades (Fig. 1). Two major clades represented *S.* Enteritidis ST11, and the other included *S.* Typhimurium and monophasic variant *S*. Typhimurium 4,[5],12:i:–. The third was a “mixed serovar” clade. The single nucleotide polymorphism (SNP) phylogeny analysis included a reference genome, *S.* Typhimurium LT2 (RefSeq accession no. NC_003197.2). Isolates with known quinolone mutations in GyrA/ParC and amino acid change positions are shown in Data Set S4.

**TABLE 3:** Sequence types determined by SISTR cgMLST and MLST 2.0^*a*^

ST determined by SISTR cgMLST	ST determined by MLST 2.0	Salmonella serovar (no. of isolates)	Total no. of isolates (*n* = 100)
ST11	ST11	*S.* Enteritidis (43)	43
ST13	ST13	*S*. Agona (1)	1
ST19	ST19	*S*. Typhimurium (11)	12
		I 4,[5],12:i:– (1)	
ST29	ST29	*S*. Stanley (2)	2
ST34	ST34	I 4,[5],12:i:– (24)	25
		*S*. Typhimurium (1)	
ST40	ST40	*S*. Derby (2)	2
ST50	ST50	*S*. Saintpaul (1)	1
ST197	ST197	*S*. Virchow (1)	1
ST198	ST198	*S*. Kentucky (1)	1
ST358	ST358	*S*. Goldcoast (1)	1
ST469	ST469	*S*. Rissen (4)	4
ST501	ST501	*S*. Singapore (1)	1
ST694	ST694	*S*. Concord (1)	1
ST1544	ST1544	*S*. Typhimurium (1)	1
ST1547	ST1547	*S*. Javiana (1)	1
ST1925	ST1925	*S.* Enteritidis (1)	1
ST2033	ST2033	*S*. Mgulani (1)	1
Unknown ST	Unknown ST	*S.* Enteritidis (1)	1

a ST, sequence type.

For the *S.* Enteritidis clade, the most common AMR gene profile was *bla*_TEM-1B_, *gyrA*, *strA*, *strB*, and *sul2* in 21 isolates (Table 4). Four isolates contained *tetA* in combination with *bla*_TEM-1B_, *gyrA*, *strA*, *strB*, *sul2*, and *tetA*, which produced the pentaresistance pattern ACSSuT (ampicillin [A], chloramphenicol [C], streptomycin [S], sulfonamide [Su], and tetracycline [T]). Only one *qnr* gene of the PMQR genes was detected in the sequence of the single *S.* Enteritidis ST11 isolate. Point mutations were identified in the QRDR of *gyrA* for all 45 *S.* Enteritidis isolates except for *S.* Enteritidis ST1925. These nucleotide changes resulted in three distinct nonsynonymous amino acid substitutions, *gyrA* D87Y (*n* = 42), D87N, and D87G (*n* = 1).

**TABLE 4:** The most common combinations of antimicrobial resistance phenotypes and genotypes in Salmonella enterica serovar Enteritidis, monophasic variant *S*. Typhimurium 4,[5],12:i:–, and *S*. Typhimurium^*a*^

Serovar	No. of antimicrobial classes	Total no. of isolates	Most common phenotypic resistance combination (no. of isolates)	Most common genotypic resistance combination (no. of isolates)
Total (*n* = 100)	0	15		
	1/2	65	AM (29)	*bla*_TEM-1B_ *gyrA strA strB sul2* (21)
				*bla*_TEM-1B_ *gyrA strA strB sul2 tetA* (4)
			AM CIP (10)	*bla*_TEM-1B_ *gyrA strA strB sul2* (10)
			AM T (9)	*bla*_TEM-1B_ *strA strB sul2 tetB* (9)
	3/4 (MDR)	20	AM CHL CIP SXT T (6)	*aadA1 aadA2 bla*_TEM-1B_ *cmlA1 dfrA12 floR qnrS1 sul2 sul3 tetA tetM* (2)


*S.* Enteritidis (*n* = 45)	0	3		
	1/2	40	AM (27)	*bla*_TEM-1B_ *gyrA strA strB sul2* (21) (ACSSu)
				*bla*_TEM-1B_ *gyrA strA strB sul2 tetA* (4) (ACSSuT)
	3/4 (MDR)	2		

Salmonella I 4,[5],12:i:– (*n* = 25)	0	0		
	1/2	15	AM T (9)	*bla*_TEM-1B_ *strA strB sul2 tetB* (9) (ASSuT)
	3/4	10	AM CIP T (2)	*bla*_TEM-1B_ *gyrA strA strB sul2 tetB* (2) (ACSSuT)
			AM CHL SXT T (2)	*aadA1 aadA2 bla*_TEM-1B_ *cmlA1 dfrA12 floR lnuF strA strB sul2 sul3 tetB* (2)

*S*. Typhimurium (*n* = 13)	0	2		
	1/2	5	AM SXT (3)	*aadA1 aadA2 bla*_TEM-1B_ *cmlA1 dfrA12 floR qnrS1 sul2 sul3 tetA tetM* (2)
	3/4 (MDR)	6	AM CHL CIP SXT T (3)	*aadA1 aadA2 bla*_TEM-1B_ *cmlA1 dfrA12 floR qnrS1 sul2 sul3 tetA tetM* (2)

a AM, ampicillin; SXT, trimethoprim-sulfamethoxazole; T, tetracycline; CHL, chloramphenicol; MDR, multidrug resistance.

In the *S*. Typhimurium clade, all 25 isolates of monophasic variant *S*. Typhimurium 4,[5],12:i:– had *tet* genes, *tetA*, *tetB*, or *tetM*, that confer resistance to tetracycline (Fig. 1). The majority had the AMR gene profile *bla*_TEM-1B_, *strA*, *strB sul2*, and *tetB*, which encoded tetraresistance to ampicillin, streptomycin, sulfonamide, and tetracycline (ASSuT) in 36% (9/25) of these strains, while two isolates had the resistance pattern ACSSuT (Table 4). Three of the four CIP-nonresistant isolates carried *qnr* genes without *gyrA*. In addition, one of the two CIP-nonresistant isolates possessed both efflux pump genes *oqxA* and *oqxB*, which confer reduced susceptibility to multiple antimicrobials, including quinolones (18, 19). Point mutations of *gyrA* gave the amino acid substitutions D87N (*n* = 1)/D87Y (*n* = 3) in ST11 and S83F (*n* = 1) in ST19.

The *S.* Typhimurium strains carried more heterogeneous AMR gene profiles and are distributed into eight different AMR genotypic profiles, with 30.8% (4/13) of the isolate genes belonging to the profile *aadA1*, *aadA2*, *bla*_TEM-1B_, *cmlA1*, *dfrA12*, *floR*, *qnrS1*, *sul2*, *sul3*, *tetA*, and *tetM* (Table 4). Five out of eight CIP-nonresistant isolates carried *qnrS* genes without any point mutation of *gyrA* or *parC*. Only one isolate of ST34 had *oqxAB* with a point mutation at *gyrA* D87Y.

For the mixed clade, almost all the serovars had point mutations in three distinct *gyrA* (*n* = 3) and *parC* (*n* = 15) regions, except for *S.* Singapore. *S.* Kentucky carried both *gyrA* and *parC* without PMQR genes.

### Clinical characteristics and Salmonella serogroups.

The characteristics of the children with gastroenteritis and the common Salmonella serogroups are listed in Table 5. Most children complained of fever (99%), diarrhea (94%), mucus (55%) or blood (35%) in stool, abdominal pain (46%), and vomiting (43%). Infants aged <24 months were more often infected by Salmonella group B (77.3%; 34/44); these include *S.* Typhimurium (29.4%; 10/34) and its monophasic variant (55.9%; 19/34) or Salmonella groups C and P, while children aged >24 months were likely infected by group D (56.5%; 26/46) predominated by *S.* Enteritidis (100.0%; 26/26) (Table S4).

**TABLE 5:** Characteristics of children with Salmonella gastroenteritis and associated serogroups (*n* = 100)^*a*^

Characteristic	Value for group				*P* value
	Total (*n* = 100)	Group B (*n* = 44)	Group D (*n* = 46)	Groups C and P (*n* = 10)	
Patient background					
No. (%) of children in age group (mo)					
<12	22	14 (31.8)	3 (6.5)	5 (50.0)	0.001*
12–<24	39	20 (45.5)	17 (37.0)	2 (20.0)	
24–<60	39	10 (22.7)	26 (56.5)	3 (30.0)	
No. (%) of children of gender					
Male	58	29 (65.9)	26 (56.5)	3 (30.0)	0.11
Female	42	15 (34.1)	20 (43.5)	7 (70.0)	

Clinical details					
No. (%) of children with:					
Diarrhea	94	44 (100.0)	41 (89.1)	9 (90.0)	0.081
Vomiting	43	16 (36.4)	21 (45.7)	6 (60.0)	0.35
Fever	99	44 (100.0)	45 (97.8)	10 (100.0)	0.55
Abdominal pain	46	23 (52.3)	20 (43.5)	3 (30.0)	0.40
Blood in stool	35	19 (43.2)	14 (30.4)	2 (20.0)	0.26
Mucus in stool	55	31 (70.5)	19 (41.3)	5 (50.0)	0.02*
Median LOS (days) (range)	3 (0–12)	3 (0–12)	3 (0–8)	3.5 (1–8)	0.34
No. (%) of children receiving intravenous rehydration	36	18 (40.9)	15 (32.6)	3 (30.0)	0.66

No. (%) of children with serovar isolated					
*S.* Enteritidis	45	0 (0.0)	45 (97.8)	0 (0.0)	<0.001*
Monophasic 4,[5],12:i:–	25	25 (56.8)	0 (0.0)	0 (0.0)	
* S*. Typhimurium	13	13 (29.5)	0 (0.0)	0 (0.0)	
Others^*b*^	17	6 (13.6)	1 (2.2)	10 (100.0)	

a LOS, length of hospital stay. *, significant at a *P* value of <0.05.

b Other serovars include *S*. Agone (*n* = 1), *S*. Concord (*n* = 1), *S*. Derby (*n* = 2), *S*. Goldcoast (*n* = 1), *S*. Javiana (*n* = 1), *S*. Kentucky (*n* = 1), *S*. Mgulani (*n* = 1), *S*. Rissen (*n* = 4), *S*. Siantpaul (*n* = 1), *S*. Singapore (*n* = 1), *S*. Stanley (*n* = 2), and *S*. Virchow (*n* = 1).

## DISCUSSION

To the best of our knowledge, this is one of the few studies using WGS to determine the serotypes and AMR of nontyphoidal Salmonella from children hospitalized for gastroenteritis in Hong Kong. This updates our epidemiology of the Salmonella serotypes and the associated AMR.

Our study showed that S. enterica serovar Typhimurium and its monophasic variant 4,[5],12:i:– were common in infants under 2 years of age, and *S.* Enteritidis was common in the older children. It is a custom of Chinese parents to feed a customized congee mixed with cooked minced pork during weaning for young infants. Raw and undercooked eggs or chicken (commonly associated with *S.* Enteritidis) is not an important source of protein until the children are older and gradually switch to an adult diet. In fact, the very high and extremely high MDR levels of *S.* Typhimurium and its monophasic variant 4,[5],12:i:– characterized by the multilocus sequence type 34 and the antimicrobial resistance profile ASSuT have been principally related to pork sources (20, 21). These clones had been previously reported in many European countries (previously described as DT194) in the early 2000s.

Salmonella species remains one of the top 10 causes of bacteremia in pediatric patients in China (22). Although bacteremia was uncommon in our cohort, a third of children complained of both fever and blood in stool, and half of the infants were under 3 months of age. MDR strains were mainly found in *S*. Typhimurium and its monophasic variant 4,[5],12:i:–. Our findings of ESBL-producing *S*. Typhimurium and *S.* Enteritidis and carbapenem-resistant *S.* Enteritidis are important in the trends of rising AMR in our community. A recent study from China demonstrated the emergence and prevalence of foodborne Salmonella harboring a chromosomally located *bla*_CTX-M-55_ gene in China (23). CTX-M-55 was the most prevalent ESBL type observed among various Salmonella Typhimurium serotypes, and the gene coexisted with plasmid-mediated fluoroquinolone resistance. It was proposed that the coselection and dissemination of ESBL and fluoroquinolone resistance in Salmonella occurred via the food chain in China (23). *bla*_CTX-M-55_ was also recently identified specifically in Salmonella spp. from pigs/pork in both Thailand and Lao PDR (24), while *bla*_CTX-M-55_ was identified in Escherichia coli from pig slaughterhouses/retail markets of these countries as well as from Cambodia and Myanmar. Further studies on animal food sources may define the widespread presence of CTX-M-55 in this region.

The overall percentage of Salmonella spp. resistant to ceftriaxone in the WHO GLASS report for Hong Kong was 5.18% in 2017 (25) and was similar to the rates of 5.9% (ceftriaxone) in Taiwan (26), 3% (ceftriaxone) in the United States (27), and 1.9% (cefotaxime) in Europe (21). None of the Salmonella spp. from Hong Kong were reported to be resistant to carbapenems (25). This is in contrast to a recent report from Zhejiang, China, of children hospitalized with NTS, with rates of resistance to ceftriaxone of 37.4% and to imipenem of 1.3% (28). However, our detection of a high prevalence of ESBL-producing *Enterobacteriaceae* of 52.8% and of carbapenem resistance from gut carriage among healthy subjects in our community in Hong Kong raises concern (29), while another study on food animal products from our wet market highlighted the high prevalence of both ESBL- and carbapenemase-producing E. coli due to NDM-1 (15). It has been noted that ESBL and carbapenem phenotypic susceptibilities are not routinely tested for extraintestinal NTS species in microbiology diagnostic laboratories, especially if first-line agents, e.g., ampicillin, fluoroquinolone, and trimethoprim-sulfamethoxazole, are susceptible according to the 2020 guidelines of the Clinical and Laboratory Standards Institute (30). The detection of the NDM gene suggests that our potential reservoir of AMR genes is much wider beyond our health care settings, and wider surveillance of AMR among Salmonella serotypes is urgently warranted.

Our study further supports the robustness of WGS for *in silico* serotyping and predicting resistance phenotypes in Salmonella enterica. It is a valuable tool that could be more widely adopted in medical and public health laboratories in order to limit the propagation and dissemination of these “superbugs.”

### Conclusion.

MDR *S*. Enteritidis, *S*. Typhimurium, and its monophasic variant were the most common serotypes causing gastroenteritis requiring hospitalization among young children. WGS-based serotyping using SISTR combines the advantages of cgMLST and AMR determination to facilitate public health surveillance. Wider surveillance of ESBL- and carbapenemase-producing Salmonella serotypes is urgently warranted.

## MATERIALS AND METHODS

### Subjects and Salmonella strains.

A case-control study of young children aged <5 years hospitalized with NTS gastroenteritis at three public hospitals (Prince of Wales Hospital, Alice Ho Miu Ling Nethersole Hospital, and United Christian Hospital) in Hong Kong was conducted between April and November 2019 (31). Of 241 children hospitalized with gastroenteritis, 101 children had laboratory-confirmed Salmonella infection, and these isolates (*n* = 101) were retrieved from the respective hospital laboratories for further analyses at the Department of Microbiology, The Chinese University of Hong Kong, Prince of Wales Hospital, Shatin, Hong Kong. Patient demographics and related data were retrieved from the electronic hospital patient records (clinical management system of the Hospital Authority of Hong Kong). Ethics approvals were granted by the Institutional Review Boards of the Joint Chinese University of Hong Kong-New Territories East Cluster Clinical Research Ethics Committee (reference no. CRE-2018.416) and the Kowloon Central/Kowloon East Research Ethics Committee (reference no. KC/KE-19-0116/ER-2).

### Serotyping and antimicrobial susceptibility testing.

Salmonella isolates were confirmed using matrix-assisted laser desorption ionization–time of flight mass spectrometry (MALDI-TOF MS) (Bruker), and strains were subjected to serotyping methods according to the White-Kauffmann-Le Minor scheme (32). Antimicrobial susceptibility testing (AST) was performed by means of the agar disk diffusion method according to CLSI guidelines (30). The susceptibilities to 9 antimicrobials (BD BBL), ampicillin (AM), cefotaxime (CTX), ceftriaxone (CRO), meropenem (MEM), tetracycline (T), chloramphenicol (CHL), trimethoprim-sulfamethoxazole (SXT), ciprofloxacin (CIP), and azithromycin (AZM), were assessed.

### DNA extraction and WGS.

DNA from cultures of the isolates grown overnight was prepared using the Wizard genomic DNA purification kit (Promega, USA), followed by DNA library preparation (Riptide DNA library preparation kit; iGenomx, USA), according to the manufacturers’ instructions and as previously described (15). WGS was performed using the NextSeq platform (Illumina, USA) from the core facility to obtain paired-end reads of 150 bp. Sequence reads were demultiplexed according to the manufacturer’s instructions. Quality control (QC) of the raw reads was performed using FastQC version 0.11.9 (http://www.bioinformatics.babraham.ac.uk/projects/fastqc/) (33) and trimmed for quality using Trimmomatic version 0.39 (http://www.usadellab.org/cms/?page=trimmomatic) (34) with the default QC setting (QC30) and the TruSeq3 Illumina adapter (TruSeq3-PE-2.fa) to remove library adapters and low-quality short reads. High-quality short reads were *de novo* assembled for contigs using SPAdes version 3.15.2 (http://cab.spbu.ru/software/spades/) (35). Contigs that aligned to Salmonella genomes by Kraken2 version 2.1.2 (http://ccb.jhu.edu/software/kraken2/) (36), were longer than 500 bp, and had coverage of >10 served as the reference index to further filter short reads using Bowtie2 version 2.4.4 (http://bowtie-bio.sourceforge.net/bowtie2/index.shtml) (37). Filtered contigs with a depth of 10 and a length of ≥500 bp from each genome were included in the analyses.

### WGS-based *in silico* serotype prediction.

Serotyping of Salmonella spp. was predicted by using WGS-assembled contig files (in FASTA format) with SeqSero1 (https://cge.cbs.dtu.dk/services/SeqSero/) (10) and with assemblies using SISTR (https://lfz.corefacility.ca/sistr-app/) (9). *In silico* 7-gene MLST sequence types (STs) were generated by the SISTR platform. The genomes were also assigned to STs using MLST 2.0 (https://cge.cbs.dtu.dk/services/MLST/) (38). Serotype predictions were compared to laboratory serogroup results using antibody agglutination. Full agreement indicates that the *in silico* method was 100% concordant with laboratory results; disagreement indicates incorrect or incongruent results of the two. No/incomplete prediction indicates that the *in silico* method was not able to provide a serogroup.

### WGS-based AMR identification.

The metadata and contig files for the Salmonella WGS data (in FASTA format) were uploaded to the CGE Bacterial Analysis Pipeline (BAP) (https://cge.cbs.dtu.dk/services/CGEpipeline-1.1) (39). AMR genes associated with phenotypes of resistance to aminoglycoside, beta-lactam, quinolone, fosfomycin, fusidic acid, glycopeptide, MLS (macrolide, lincosamide, and streptogramin B), nitroimidazole, oxazolidinone, phenicol, rifampicin, sulfonamide, tetracycline, and trimethoprim (40) were searched by a BLAST analysis (41) and mapped to CGE ResFinder v2.1 with a 90% identify threshold and a 60% minimum coverage length. Known mutations in the *gyrA*, *gyrB*, *parA*, *parB*, *parC*, and *parE* genes that confer quinolone resistance (42) were identified using CGE PointFinder (now part of ResFinder) for chromosomal and known SNP mutations (43).

### SNP phylogenetic tree.

SNP analysis of Salmonella was conducted using the CGE CSI Phylogeny 1.4 pipeline (https://cge.cbs.dtu.dk/services/CSIPhylogeny/) (44). Reads were mapped to the publicly available complete genome of Salmonella Typhimurium strain LT2 (RefSeq accession no. NC_003197.2) as a reference. The SNP maximum likelihood tree (Newick file) was visualized with iTOL v.3 (45).

### Statistical analysis.

Patient demographic and laboratory data were analyzed using SPSS v22.0. Categorical data were analyzed using a chi-square test and Fisher’s exact test. A Kruskal-Wallis test was used for nonnormally distributed data that were presented as medians and interquartile ranges [IRs]. The Salmonella serotypes recognized by SISTR were grouped into multidrug-resistant (MDR) (resistant to ≥3 drugs) and non-multidrug-resistant (resistance to <3 antimicrobial agents) serotypes. Statistical significance was considered a *P* value of <0.05.

### Data availability.

All sequence data associated with this study have been deposited in the NCBI database under BioProject accession no. PRJNA678820.
