# Supplementary material for: Genomic Epidemiology of Multidrug-Resistant Nontyphoidal Salmonella in Young Children Hospitalized for Gastroenteritis
Source: Microbiol Spectr. 2021 Aug 4;9(1):10.1128/spectrum.00248-21. doi: 10.1128/spectrum.00248-21 (PMC8552638; doi:10.1128/spectrum.00248-21)
Supplement: SUPPLEMENTAL FILE 2 — Supplemental material. Download SPECTRUM00248-21_Supp_1_seq5.pdf, PDF file, 0.2 MB [file spectrum00248-21_supp_1_seq5.pdf]

Table S1 Summary of *Salmonella* serovars predictions by laboratory, SISTR and SeqSero1

Platforms	Predicted serogroup	Predicted serotypes	Antigenic formula	Number of isolates	Total (%)
Laboratory (n=100)	B	Typhimurium	-	7	46
		N/A	-	39	
	C	N/A	-	8	8
	D	Enteritidis	-	15	46
		N/A	-	31	
SISTR (n=100)	B	I 4,[5],12:i:-	4:i:-	25	44
		Typhimurium	4:i:1,2	13	
		Derby	4:f,g:-	2	
		Stanley	4:d:1,2	2	
		Agona	4:f,g,s:-	1	
		Saintpaul	4:e,h:1,2	1	
	C	Rissen	7:f,g:-	4	9
		Virchow	7:r:1,2	1	
		Singapore	7:k:e,n,x	1	
		Kentucky	8:i:z6	1	
		Goldcoast	8:r:l,w	1	
		Concord	7:l,v:1,2	1	
	D	Enteritidis	9:g,m:-	45	46
		Javiana	9:l,z28:1,5	1	
	P	Mgulani	38:i:1,2	1	1
SeqSero1 (n=100)	B	I 4,[5],12:i:-	4:i:-	26	44
		Typhimurium	4:i:1,2	11	
		Derby	4:f,g:-	2	
		Stanley	4:d:1,2	1	
		Agona	4:f,g,s:-	1	
		N/A	4:d:-	1	
		N/A	4:-:1,2	1	
		N/A	4:-:-	1	
	C	Rissen	7:f,g:-	4	9
		Virchow	7:r:1,2	1	
		Singapore	7:k:e,n,x	1	
		Kentucky	8:i:z6	1	
		Goldcoast or Brikama*	8:r:l,w*	1	
		N/A	7:-:1,2	1	
	D	Enteritidis	9:g,m:-	45	46
		II 9,12:l,z28:1,5 or Javiana*	9:l,z28:1,5*	1	
	P	N/A	38:-:1,2	1	1

* Multiple serovar predictions sharing the same antigenic formula

N/A- Not predicted

Table S2 Disagreement of serogroup O antigens matches by SISTR and SeqSero1 compared to laboratory serotyping

Isolate ID	Predicted serogroup			Disagreements	Description
	Laboratory	SISTR	SeqSero1		
A013_06/19	B	P	P	Incorrect result	incorrect calling of O antigenic determinants with respect to traditional serotyping
A084_08/19	B	C	C	Incorrect result	incorrect calling of O antigenic determinants with respect to traditional serotyping
A087_08/19	N/A	B	B	Incongruent result	non-expressed of O antigenic determinant phenotypically

N/A- Not predicted.

Table S3 Resistance genes and resistance phenotype detected in the 100 *Salmonella* isolates by ResFinder 2.1

Resistance genes	Resistance phenotype	Total (n=100)	Number of isolates (n)														
			Agona (n=1)	Concord (n=1)	Derby (n=2)	Enteritidis (n=45)	Goldcoast (n=1)	I 4,[5],12:i:- (n=25)	Javiana (n=1)	Kentucky (n=1)	Mgulani (n=1)	Rissen (n=4)	Saintpaul (n=1)	Singapore (n=1)	Stanley (n=2)	Typhimurium (n=13)	Virchow (n=1)
**Aminoglycoside**																	
strA	STR	62	-	-	-	39	-	22	-	-	-	-	-	-	-	1	-
strB	STR	62	-	-	-	39	-	22	-	-	-	-	-	-	-	1	-
aadA5	STR	2	-	-	-	1	-	-	-	-	-	-	-	-	-	1	-
aadA7	STR	1	-	-	-	-	-	-	-	1	-	-	-	-	-	-	-
aadA16	STR	1	-	-	-	-	-	-	-	-	-	-	-	-	-	1	-
aadA17	STR	1	-	-	-	-	-	1	-	-	-	-	-	-	-	-	-
aadA1	STR	20	-	-	1	-	1	7	-	-	-	2	-	-	-	9	-
aadA2	STR	22	-	-	1	1	1	8	-	-	-	2	-	-	-	9	-
aadA24	STR	2	-	-	-	-	-	2	-	-	-	-	-	-	-	-	-
aac(3)-IVa	GEN	94	1	1	2	44	1	21	1	1	1	4	1	1	2	12	1
aac(3)-IId	GEN	2	-	-	-	-	-	-	-	-	-	-	-	-	-	2	-
aac(3)-Id	GEN	1	-	-	-	-	-	-	-	1	-	-	-	-	-	-	-
aph(3')-Ia	NEO	2	-	-	-	-	-	1	-	-	-	-	-	-	-	1	-
aph(3')-Ic	NEO	3	-	-	-	1	-	2	-	-	-	-	-	-	-	-	-
aph(4)-Ia	HGY B	6	-	-	-	1	-	4	-	-	-	-	-	-	-	1	-
**Quinolone**																	
aac(6')lb-cr	CIP	2	-	-	-	-	-	1	-	-	-	-	-	-	-	1	-
qnrS1	CIP	15	-	-	1	1	1	4	-	-	-	-	-	-	-	8	-
oqxA	CIP	3	-	-	-	-	-	2	-	-	-	-	-	-	-	1	-
oqxB	CIP	3	-	-	-	-	-	2	-	-	-	-	-	-	-	1	-
gyrA	CIP	52	-	-	-	44	-	5	-	1	-	-	1	-	-	1	-
parC	CIP	15	1	1	2	-	1	-	1	1	1	4	-	-	2	-	1
**Sulphanomide**																	
sul1	SMX	6	-	-	-	-	-	1	-	1	-	2	-	-	-	2	-
sul2	SMX	74	-	-	-	39	1	24	-	-	-	-	-	-	-	10	-
sul3	SMX	20	-	-	-	1	1	8	-	-	-	2	-	-	-	8	-
**Trimethoprim**																	
dfrA27	SXT	1	-	-	-	-	-	-	-	-	-	-	-	-	-	1	-
dfrA17	SXT	2	-	-	-	1	-	-	-	-	-	-	-	-	-	1	-
dfrA14	SXT	2	-	-	-	-	-	2	-	-	-	-	-	-	-	-	-
dfrA12	SXT	17	-	-	-	-	1	6	-	-	-	2	-	-	-	8	-
**Tetrcycline**																	
tet(A)	T	25	-	-	1	7	1	2	-	1	-	4	-	-	-	9	-

tet(B)	T	26	-	-	-	-	-	25	-	-	-	-	-	-	-	1	-
tet(M)	T	12	-	-	1	-	1	1	-	-	-	-	-	-	-	9	-
**Phenicol**																	
catB3	CHL	2	-	-	-	-	-	1	-	-	-	-	-	-	-	1	-
cmlA1	CHL	23	-	-	1	1	1	9	-	-	-	2	-	-	-	9	-
floR	CHL	21	-	-	-	2	1	8	-	-	-	-	-	-	-	10	-
**Beta-lactam**																	
blaTEM-1B	AM	73	-	-	-	40	1	21	-	1	-	2	-	-	-	8	-
blaOXA-10	AM	3	-	-	-	1	-	2	-	-	-	-	-	-	-	-	-
blaOXA-1	AM	1	-	-	-	-	-	1	-	-	-	-	-	-	-	-	-
blaNDM-1	AM, CTX, CRO, MEM	1	-	-	-	-	-	-	-	-	-	-	-	-	-	1	-
blaCTX-M-55	AM, CTX, CRO	4	-	-	-	1	-	2	-	-	-	-	-	-	-	1	-
blaCTX-M-64	AM, CTX, CRO	1	-	-	-	1	-	-	-	-	-	-	-	-	-	-	-
blaCTX-M-65	AM, CTX, CRO	1	-	-	-	-	-	1	-	-	-	-	-	-	-	-	-
**Rifampicin**																	
ARR-2	RMP	3	-	-	-	1	-	2	-	-	-	-	-	-	-	-	-
ARR-3	RMP	2	-	-	-	-	-	1	-	-	-	-	-	-	-	1	-
**Macrolide**																	
mph(A)	AZM	1	-	-	-	-	-	-	-	-	-	-	-	-	-	1	-
**Lincosamide**																	
lnu(F)	LINC	8	-	-	-	1	-	7	-	-	-	-	-	-	-	-	-
**Colistin**																	
mcr-1	CST	1	-	-	-	1	-	-	-	-	-	-	-	-	-	-	-

STR-Streptomycin; GEN-Gentamycin; NEO-Neomycin; HGY B-Hygromycin B; CIP-Ciprofloxacin; SMX-Sulfamethoxazole; SXT-Trimethoprim-Sulfamethoxazole; T-Tetracycline; CHL-Chloramphenicol; AM-Ampicillin; CTX-Cefotaxime; CRO-Cetriaxone; MEM-Meropenem; RMP- Rifampicin; AZM-Azithromycin; LINC-Lincomycin; CST-Colistin.

Table S4 Distribution of nontyphoidal *Salmonella* (n=100) by patient's age

**Serogroup**	**Serovars**	**Child's age (months)**			**Total**
		**<12m**	**12 to <24m**	**24 to <60m**	**n**
		**n (%)**	**n (%)**	**n (%)**	
Total patients		22	39	39	100
D	*S.* Enteritidis	3 (13.6)	16 (41.0)	26 (66.7)	45
B	*S.* Typhimurium	2 (9.1)	8 (20.5)	3 (7.7)	13
B	*S.* I 4,[5],12:i:-	10 (45.5)	9 (23.1)	6 (15.4)	25
Others *Salmonella* spp.					
B	*S.* Agona	0 (0)	0 (0)	1 (2.6)	1
C	*S.* Concord	0 (0)	1 (2.6)	0 (0)	1
B	*S.* Derby	2 (9.1)	0 (0)	0 (0)	2
C	*S.* Goldcoast	1 (4.5)	0 (0)	0 (0)	1
D	*S.* Javiana	0 (0)	1 (2.6)	0 (0)	1
C	*S.* Kentucky	0 (0)	0 (0)	1 (2.6)	1
P	*S.* Mgulani	1 (4.5)	0 (0)	0 (0)	1
C	*S.* Risen	3 (13.6)	1 (2.6)	0 (0)	4
B	*S.* Saintpaul	0 (0)	1 (2.6)	0 (0)	1
C	*S.* Singapore	0 (0)	0 (0)	1 (2.6)	1
B	*S.* Stanley	0 (0)	2 (5.1)	0 (0)	2
C	*S.* Virchow	0 (0)	0 (0)	1 (2.6)	1
